# Functional regression method for whole genome eQTL epistasis analysis with sequencing data

**DOI:** 10.1186/s12864-017-3777-4

**Published:** 2017-05-18

**Authors:** Kelin Xu, Li Jin, Momiao Xiong

**Affiliations:** 1 State Key Laboratory of Genetic Engineering and Ministry of Education Key Laboratory of Contemporary Anthropology, Collaborative Innovation Center for Genetics and Development, School of Life Sciences, Fudan University, Shanghai, 200438 China; 2 School of Data Science and Institute for Big Data, Fudan University, Shanghai, 200433 China; 3 Department of Biostatistics, Human Genetics Center, The University of Texas Health Science Center at Houston, Houston, TX 77030 USA; 4 Human Genetics Center, The University of Texas Health Science Center at Houston, P.O. Box 20186, Houston, TX 77225 USA

**Keywords:** Gene-gene interaction, Multivariate functional regression, Functional regression models, RNA-seq, Next-generation sequencing, Association studies, eQTL

## Abstract

**Background:**

Epistasis plays an essential rule in understanding the regulation mechanisms and is an essential component of the genetic architecture of the gene expressions. However, interaction analysis of gene expressions remains fundamentally unexplored due to great computational challenges and data availability. Due to variation in splicing, transcription start sites, polyadenylation sites, post-transcriptional RNA editing across the entire gene, and transcription rates of the cells, RNA-seq measurements generate large expression variability and collectively create the observed position level read count curves. A single number for measuring gene expression which is widely used for microarray measured gene expression analysis is highly unlikely to sufficiently account for large expression variation across the gene. Simultaneously analyzing epistatic architecture using the RNA-seq and whole genome sequencing (WGS) data poses enormous challenges.

**Methods:**

We develop a nonlinear functional regression model (FRGM) with functional responses where the position-level read counts within a gene are taken as a function of genomic position, and functional predictors where genotype profiles are viewed as a function of genomic position, for epistasis analysis with RNA-seq data. Instead of testing the interaction of all possible pair-wises SNPs, the FRGM takes a gene as a basic unit for epistasis analysis, which tests for the interaction of all possible pairs of genes and use all the information that can be accessed to collectively test interaction between all possible pairs of SNPs within two genome regions.

**Results:**

By large-scale simulations, we demonstrate that the proposed FRGM for epistasis analysis can achieve the correct type 1 error and has higher power to detect the interactions between genes than the existing methods. The proposed methods are applied to the RNA-seq and WGS data from the 1000 Genome Project. The numbers of pairs of significantly interacting genes after Bonferroni correction identified using FRGM, RPKM and DESeq were 16,2361, 260 and 51, respectively, from the 350 European samples.

**Conclusions:**

The proposed FRGM for epistasis analysis of RNA-seq can capture isoform and position-level information and will have a broad application. Both simulations and real data analysis highlight the potential for the FRGM to be a good choice of the epistatic analysis with sequencing data.

**Electronic supplementary material:**

The online version of this article (doi:10.1186/s12864-017-3777-4) contains supplementary material, which is available to authorized users.

## Background

Epistatic effect in gene expression, defined as the departure from additive effects in a linear model of eQTL analysis [1], plays an essential role in understanding the gene regulation and disease mechanisms [2–4]. One polymorphism’s effect on expression of a gene depends on other polymorphisms present in the genome [5]. Epistasis analysis of gene expressions will substantially improve the understanding of the genetic architecture of gene expression and facilitate mechanistic insights into complex traits [6, 7]. However, eQTL epistasis analysis remains fundamentally unexplored due to large computational challenges and data availability [6].

Gene expression is an intermediate phenotype that bridges the genotype and higher level phenotypes such as diseases [8, 9]. Studying the effect of epistasis on the gene expression could provide a better understanding of the genetic architecture and gene regulation. The importance of detecting the epistatic effect on the gene expression has been emphasized in many recent studies [10, 11]. However, the corresponding methods are relatively rare. The widely used statistical methods for identifying eQTL epistasis are designed for microarray expression data where an overall expression of the gene is taken as a quantitative trait and all methods for QTL epistasis analysis can be used for eQTL epistasis analysis [10, 12].

Application of next generation sequencing (NGS) techniques to the genetic analysis of gene expression involves (1) generating millions of short reads of mRNA or cDNA which are mapped to the genome and lead to a sequence of read counts at the hundreds of millions of genomic positions [13–16] and (2) generating millions or 10 millions of genetic variants. RNA-seq counts vary greatly across the gene [17]. Count variations can be due to experimental bias such as fragmentation methods, reverse-transcription [16], sequence-specific bias and sequencing technology variation [18]. However, count variation can also be caused by variation in splicing, transcription start sites, polyadenylation sites, post-transcriptional RNA editing across the entire gene, and transcription rates of the cells [13–16, 18]. RNA-seq data can be viewed as a function or a curve of the genomic position and hence can be taken as a function-valued trait.

Although RNA-seq data are measured as a function, the widely used methods for genetic studies of the RNA-seq in humans are the same as that for the traditional single-valued quantitative traits where a single number for overall expression of the gene is taken as a quantitative trait. These methods use summary statistics to measure or represent gene expressions assayed by NGS techniques and cannot capture the expression variations across the gene due to splicing, transcription start sites, polyadenylation sites, post-transcriptional RNA editing across the entire gene, and transcription rates of the various cells. The summary statistic-based epistasis analysis of the RNA-seq fails to utilize all transcripts information.

The critical barrier in epistasis analysis is to deal with rare variants. The traditional statistical methods for epistasis analysis were originally designed for testing the interaction between common variants and are difficult to apply to rare variants due to high type 1 error rates, severe multiple testing, prohibitive computational time and low power [19]. Whole genome RNA-req eQTL analysis poses a significant challenge. To meet the challenge, we developed a nonlinear functional regression model (FRGM) with functional responses where the position-level read counts within a gene are taken as a function of genomic position, and functional predictors where genotype profiles are viewed as a function of genomic position, for epistasis analysis with RNA-seq data, which allows simultaneous capture of all space information hidden in the RNA-seq data and genetic variation data, but with substantially reduced dimensions. Instead of testing the interaction of all possible pair-wises SNPs, the FRGM takes a gene as a basic unit for epistasis analysis, which tests for the interaction of all possible pairs of genes and uses all the information that can be accessed to collectively test interaction between all possible pairs of SNPs within two genome regions (or genes). The proposed FRGM for epistasis analysis of the RNA-seq can capture isoform and position-level information and will have a broad application.

The FRGM for epistasis analysis has several remarkable features. First, the FRGM accounts for the change in the position-level read counts, while preserving the intrinsic structure and all the positional-level genetic information. Second, the FRGM simultaneously utilizes both correlation information among the RNA-seq at different genomic positions and among all variants in a genomic region. Third, the multicollinearity problems in the FRGM which may be presented in both the RNA-seq and genetic variation are alleviated. Fourth, the FRGM expands both position-level read count function and genotype function in terms of orthogonal eigenfunction, which leads to substantial dimension reduction in both RNA-seq data and SNP data. The FRGM for epistasis analysis of function-valued traits which capture key information in the data is expected to open a new route for epistasis analysis of RNA-seq data.

To evaluate its performance for epistasis analysis of the RNA-seq, we use large scale simulations to calculate the type I error rates and evaluate the power of the proposed FRGM for detecting epistasis. To further evaluate its performance, the FRGM for epistasis analysis is applied to 350 samples with both RNA-seq and NGS data from the 1000 Genomes Project. An R packge for implementing the developed FRGM for epistasis analysis of RNA-seq and NGS data can be downloaded from our website https://sph.uth.edu/research/centers/hgc/xiong/software.htm.

## Results

### Null distribution of test statistics

To examine the null distribution of test statistics, we performed a series of simulation studies to compare their empirical levels with the nominal ones. We consider three models for type 1 error rate simulations: model 1 with no marginal effects, model 2 with marginal effects at the first gene and model 3 with marginal effects at both the first and second genes.

We generated 100,000 chromosomes by resampling from the 350 European samples with genetic variants in five genes*: IRAK3, ACSS3, SUV420H1, ETV7*, and *HPS4* from the next generation sequencing data in the 1000 Genomes Project. The summary statistics of the variants in five genes are summarized in Additional file 1: Table S1. The marginal genetic effects will be estimated from the data. 100 genes with RNA-seq data were randomly selected from GEUVADIS project. They were used to develop the models for generating RNA-seq data in simulation (Detailed description were referred to Method Section).10 pairs of genes were selected from five genes : IRAK3, ACSS3, SUV420H1, ETV7, and HPS4 with genotype data from 1000 Genome Project dataset.

The number of sampled individuals from the population ranged from 1000 to 5,000, and 5,000 simulations were repeated. We randomly selected 10% of the SNPs as causal variants from five genes: *IRAK3, ACSS3, SUV420H1, ETV7*, and *HPS4*. We perfume gene-gene interaction tests for 10 pairs of genes selected from five genes with genotypes under the three models for 5000 times. The type 1 error rates were averaged over 10 pairs of genes with genotype data and 5,000 simulations for each model. Tables 1, 2 and 3 summarized the type I error rates of the test statistics for testing the interaction between two genes with no marginal effect, marginal effect at the first gene and marginal effects at both genes consisting only of rare variants and both common and rare variants, respectively, averaged over 100 genes with RNA-seq data and 10 pairs of genes with genotype data at the nominal levels α = 0.05, α = 0.01 and α = 0.001. These results clearly showed that the type I error rates of the FRGM-based test statistics for testing interaction between two genes with or without marginal effects were not appreciably different from the nominal *α* levels.

**Table 1:** Average type 1 error rates of the statistic for testing interaction between two genes with no marginal effect over 10 pairs of genes

	Rare Variants			Common & Rare Variants		
Sample Size	0.05	0.01	0.001	0.05	0.01	0.001
1000	0.0495	0.0099	0.0009	0.0497	0.0101	0.0010
2000	0.0501	0.0094	0.0010	0.0510	0.0100	0.0011
3000	0.0475	0.0097	0.0011	0.0498	0.0103	0.0011
4000	0.0497	0.0097	0.0011	0.0501	0.0101	0.0009
5000	0.0499	0.0104	0.0011	0.0511	0.0108	0.0010

**Table 2:** Average type 1 error rates of the statistic for testing interaction between two genes with marginal effect at the first genes over 10 pairs of genes

Rare Variants			Common & Rare Variants		
0.05	0.01	0.001	0.05	0.01	0.001
0.0507	0.0094	0.0007	0.0491	0.0100	0.0010
0.0500	0.0098	0.0009	0.0489	0.0099	0.0012
0.0508	0.0108	0.0010	0.0496	0.0096	0.0011
0.0490	0.0101	0.0010	0.0498	0.0103	0.0011
0.0490	0.0101	0.0010	0.0506	0.0093	0.0008

**Table 3:** Average type 1 error rates of the statistic for testing interaction between two genes with marginal effects at two genes over 10 pairs of genes

	Rare Variants			Common & Rare Variants		
Sample Size	0.05	0.01	0.001	0.05	0.01	0.001
1000	0.0501	0.0108	0.0011	0.0486	0.0103	0.0010
2000	0.0493	0.0098	0.0010	0.0495	0.0101	0.0010
3000	0.0499	0.0095	0.0011	0.0497	0.0101	0.0011
4000	0.0494	0.0099	0.0010	0.0496	0.0096	0.0008
5000	0.0489	0.0097	0.0011	0.0497	0.0107	0.0010

### Power evaluation

To evaluate the performance of the functional regression model for testing the epistatic effect on gene expression, we estimated the power through simulations. We generated 100,000 chromosomes by resampling from the 350 European samples with genetic variants in two genes: IRAK3 and ACSS3 from the next generation sequencing data in 1000 Genomes Project. We randomly selected 20% variants as causal variants, assumed that there were *k*_*1*_ SNPs in the first gene, and *k*_*2*_ SNPs in the second gene. Two thousand individuals were sampled. We assumed that both marginal effects and epistasis effects were a function of the genomic position and used the multiple regression models to generate the RNA-seq data under four interaction models: Dominant OR Dominant, Dominant AND Dominant, Recessive OR Recessive and Threshold (See the Methods section).

We compared the power of the FRGM with both functional response and functional predictors (BFGM), FRGM with scalar response and functional predictors (SFGM) and regression on principal component analysis (PCA). For the PCA method, the PCA was performed on the RNA-seq data and the number of PCs were selected to explain 80% variance of number of reads at different genomic positions. The multiple functional regression was performed to analyze the data [20]. In the BFGM, both RNA-seq and genotype profiles were taken as a function of genomic position and expanded in terms of functional principal components.

**Fig. 1:**
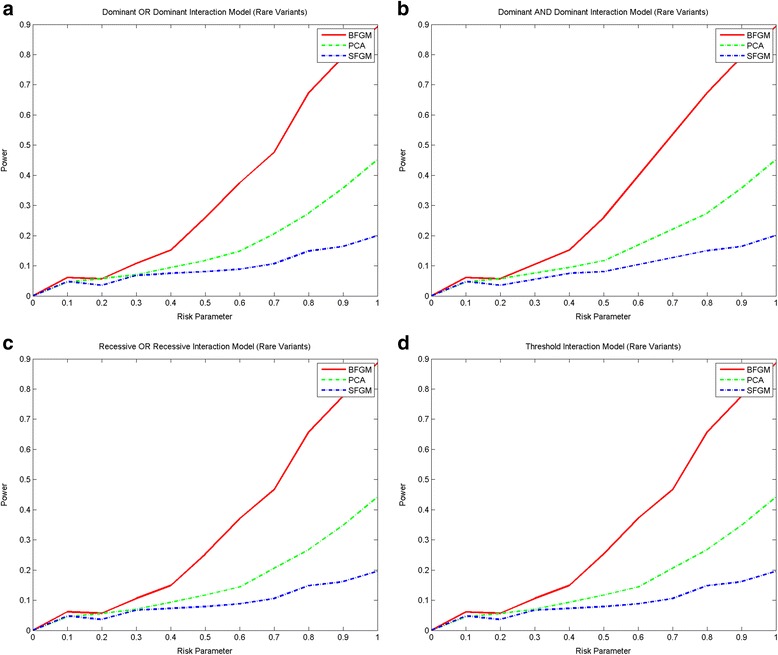
**a**. Power curves of three statistics: the BFGM, regression on PCA, SFGM, for testing interaction between two genomic regions that consist of rare variants with the RNA-seq trait as a function of the relative risk parameter *r* at the significance level *α* = 0.05 under the Dominant OR Dominant model, assuming sample sizes of 2,000. **b**. Power curves of three statistics: the BFGM, regression on PCA, SFGM, for testing interaction between two genomic regions that consist of rare variants with RNA-seq trait as a function of the relative risk parameter *r* at the significance level *α* = 0.05 under the Dominant AND Dominantmodel, assuming sample sizes of 2,000. **c**. Power curves of three statistics: the BFGM, regression on PCA, SFGM, for testing interaction between two genomic regions that consist of rare variants with RNA-seq trait as a function of the relative risk parameter *r* at the significance level *α* = 0.05 under the Recessive OR Recessive model, assuming sample sizes of 2,000. **d**. Power curves of three statistics: the BFGM, regression on PCA, SFGM, for testing interaction between two genomic regions that consist of rare variants with RNA-seq trait as a function of the relative risk parameter *r* at the significance level *α* = 0.05 under the Threshold model, assuming sample sizes of 2,000

Figure 1a-d plotted the power curves of three statistics: BFGM, SFGM and PCA to test the interaction between two genes with rare variants under the Dominant OR Dominant, Dominant AND Dominant, Recessive OR Recessive and Threshold models, respectively. In the simulation, 20% of the rare variants were randomly selected as the causal variants. These power curves were a function of the risk parameter at the significance level *α* = 0.05. We observed that under all four interaction models the BFGM had the highest power, followed by the regression on PCA. Power of the SFGM was the lowest. The results demonstrated that summary statistics such as RPKM for measuring gene expression could not capture the expression variations across the gene and almost had no power to detect the interaction between two genes with rare variants.

**Fig. 2:**
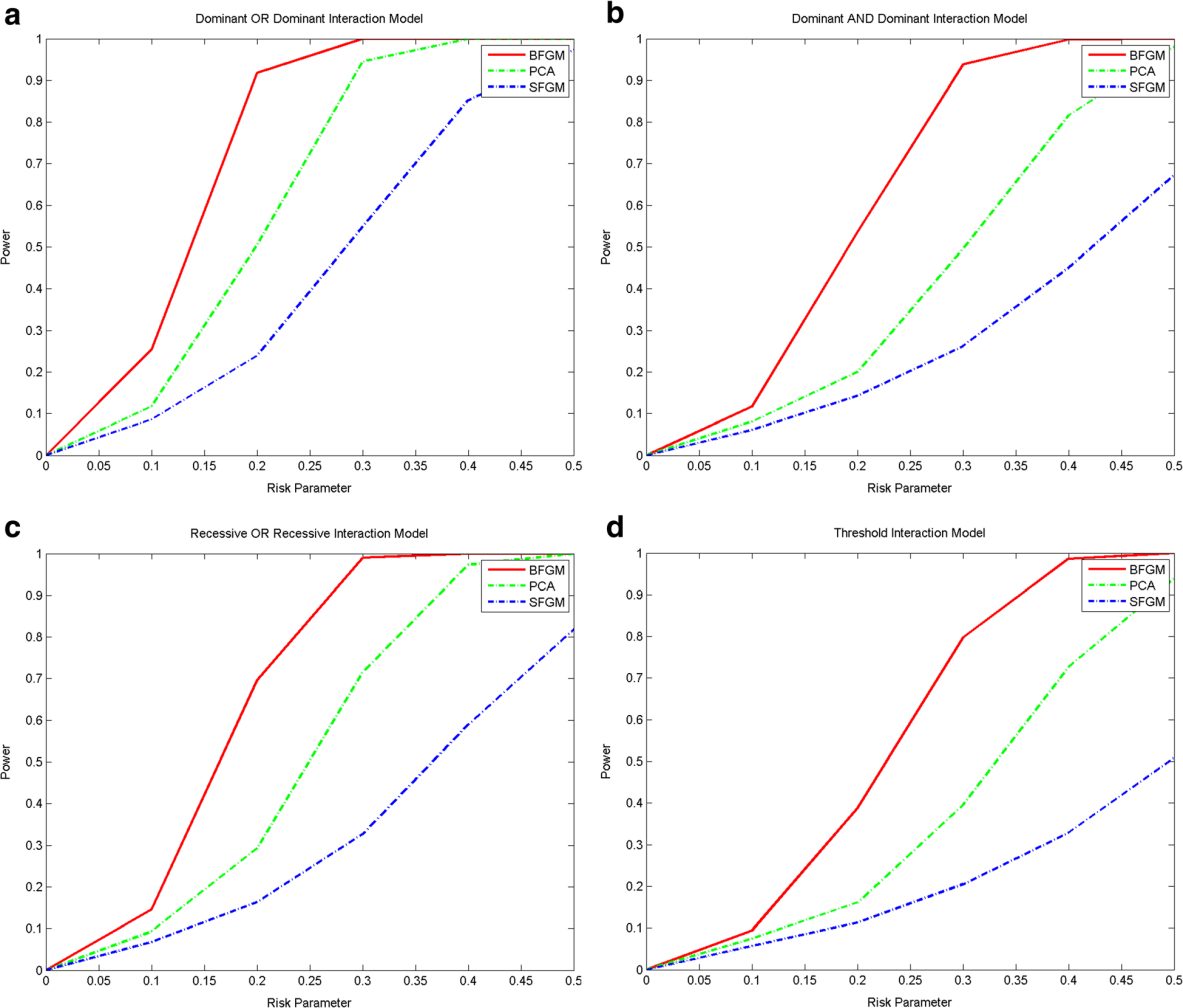
**a**. Power curves of three statistics: the BFGM, regression on PCA, SFGM, for testing interaction between two genomic regions that consist of both common and rare variants with the RNA-seq trait as a function of the relative risk parameter *r* at the significance level *α* = 0.05 under the Dominant OR Dominant model, assuming sample sizes of 2,000. **b**. Power curves of three statistics: the BFGM, regression on PCA, SFGM, for testing interaction between two genomic regions that consist of both common and rare variants with RNA-seq trait as a function of the relative risk parameter *r* at the significance level *α* = 0.05 under the Dominant AND Dominantmodel, assuming sample sizes of 2,000. **c**. Power curves of three statistics: the BFGM, regression on PCA, SFGM, for testing interaction between two genomic regions that consist of both common and rare variants with RNA-seq trait as a function of the relative risk parameter *r* at the significance level *α* = 0.05 under the Recessive OR Recessive model, assuming sample sizes of 2,000. **d**. Power curves of three statistics: the BFGM, regression on PCA, SFGM, for testing interaction between two genomic regions that consist of both common and rare variants with RNA-seq trait as a function of the relative risk parameter *r* at the significance level *α* = 0.05 under the Threshold model, assuming sample sizes of 2,000

The BFGM can also be applied to the presence of both common and rare variants. Figure 2a-d plotted the power curves of three statistics for testing interaction between two genes with both common and rare variants where 10% of the common variants and 10% of the rare variants were chosen as causal variants under the Dominant OR Dominant, Dominant AND Dominant, Recessive OR Recessive and Threshold models, respectively. The power patterns of tests for the interactions between two genes with both common and rare variants were similar to that with rare variants only. The BFGM had the highest power, followed by the PCA and the SFGM. However, we noticed that the power of the SFGM for epistasis analysis in the presence of common variants increased substantially. Under some models such as the Dominant OR Dominant model, the SFGM would have enough power to detect interactions between two genes with common variants.

### RNA-seq data and NGS data

The BFGM was applied to the RNA-seq data in the GEUVADIS RNA Sequencing Project [21] and the WGS data in the1000 Genomes Project. A total of 350 samples with European origin was shared between the GEUVADIS RNA Sequencing Project and 1000 Genomes Project, which had combined transcriptome (22,706 gene expressions measured by RNA-seq) and genome sequencing data (2,708,453 SNPs in 24,519 genes). After removing singleton SNPs, repeated SNPs, and filtering out the SNPs violating HW equilibrium [22] (*P* value < 10^−9^ for declaring HW disequilibrium), 2,566,261 SNPs in 18,986 genes were included in the epistasis analysis. In the RNA-seq data pre-processing, we removed the genes whose expressing rates were less than 30% and the genes that did not contain any SNPs. Finally, RNA-seq data of the 15,656 genes were included in the analysis. We used DESeq [23] to normalize the RNA-seq data.

### Cis-trans interactions

**Fig. 3:**
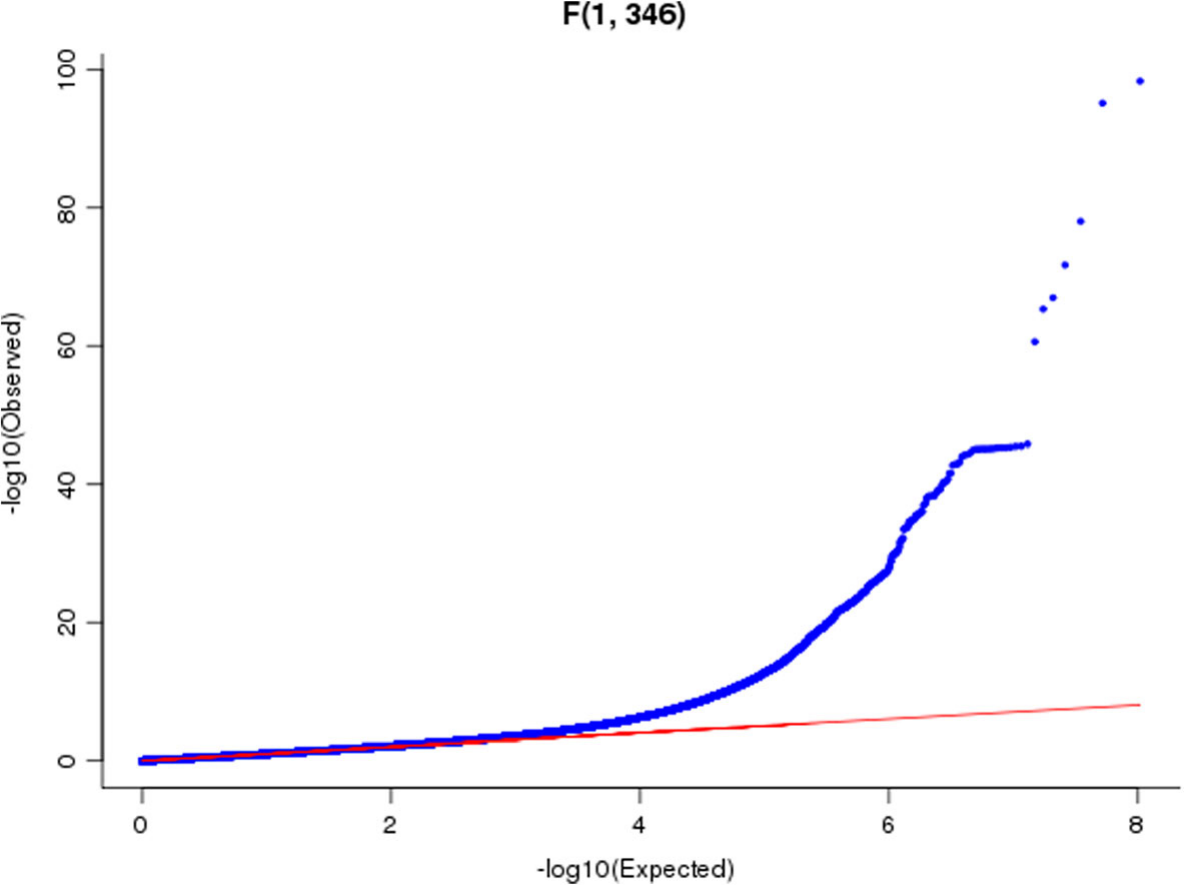
QQ plot of P-values from the BFGM for testing the *cis*-trans interactions between two genes influencing transcription

We considered the RNA-seq curve of the target gene as a function-valued trait. The target gene selected from the 15656 gene expressions was referred to as gene 1. We selected one of the remaining 18985 genotyping genes as gene 2. We used BFGM to test for the interactions between gene 1 and gene 2 influencing the expression of the target gene 1. The total number of gene pairs tested for interactions which included both common and rare variants was 297,229,160. A *P*-value for declaring significant interaction after applying the Bonferroni correction for multiple tests was 1.68 × 10^−10^. To examine the behavior of the BFGM, we plotted the QQ plot of the test (Fig. 3). QQ plot showed that the false positive rate of the BFGM for detection of epistasis was controlled.

**Fig. 4:**
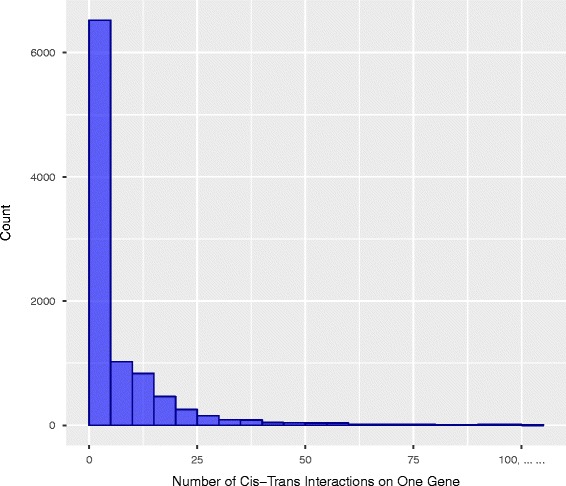
A histogram showing a distribution of the number of cis-trans gene-gene interactions on each gene expression

For comparisons, the SFGM was also applied to the dataset. RPKM and DESeq were used to compute the overall expression value of genes from the RNA-seq data. All the expression values were processed by the rank-based inverse normal transformation [24]. For both common and rare variants, in total, 162361, 260 and 51 significant *cis*-trans interactions regulating the gene expressions were identified by the BFGM, SFGM with the RPKM and DESeq, respectively. We observed 9,846genes whose expressions were influenced by 16,2361*cis-*trans interactions. We found that the average number of epistasis influencing each gene was 16. A total of 3,505 gene expressions were influenced by one significant *cis*-trans gene-gene interactions, 169 gene expressions were influenced by more than 100 *cis*-trans gene-gene interactions. Figure 4 presented a histogram that showed a distribution of the *cis*-trans gene-gene interactions.

The *P*-values of the top 20 interactions between genes ranked by the BFGM method were summarized in Table 4 where *P*-values for testing interactions between genes by the SFGM (RPKM, DESeq and RNAmin) and min *P*-values were also listed. The RNAmin denoted the minimum of *P*-values computed by the SFGM method with the number of reads at each genome position of the gene as the scalar response in the functional regression model. The min *P*-values denoted to take the minimum of all *P*-values for testing all possible pairs of SNPs between two genes using functional regression model with functional response and scalar predictors. Table 4 showed several remarkable features. First, we often observed the pair-wise interaction between rare and rare variants (34.38%), and rare and common variants (59.38%). Less observed was the significant pair-wise interaction between common and common variants (6.25%). Second, significant interactions between two genes often indicated that at least one significant pair of SNPs in two genes could be observed (min *P*-values were small). However, we can observe that pairs of SNPs between two genes jointly had significant interaction effects, but individually each pair of SNPs mildly contributed to the interaction effects. Third, the BFGM often had a much smaller *P*-value to detect interaction than other tests. Fourth, we observed that genes may not show even mild marginal association, but they did demonstrate significant evidence of interaction. If only the interactions between two marginally significant genes are tested, some significant interactions may be missed. The fifth, the BFGM tremendously reduced computation burden.

**Table 4:** *P*-values of top 20 genes ranked by the BFGM methods

							*P*-value (Interaction)				
								SFGM			
Gene Expression	Gene 1	Chr	Marginal (eQTL)	Gene 2	Chr	Marginal (eQTL)	BFGM	RPKM	DESeq	RNA-min	min *P*-value
ULK4	ULK4	3	1.45E-01	C19orf70	19	7.96E-06	0.00E + 00	4.17E-01	4.98E-01	0.00E + 00	0.00E + 00
ULK4	ULK4	3	1.45E-01	OR10A2	11	6.04E-15	4.07E-305	4.33E-05	4.97E-03	0.00E + 00	0.00E + 00
CCDC13	CCDC13	3	5.60E-01	TMEM121	14	9.94E-24	2.91E-302	4.98E-03	1.73E-02	1.36E-304	2.12E-12
ULK4	ULK4	3	1.45E-01	PSMC5	17	7.30E-23	5.72E-267	2.79E-05	1.50E-03	0.00E + 00	0.00E + 00
ULK4	ULK4	3	1.45E-01	COX5B	2	1.15E-03	2.46E-242	1.82E-03	3.06E-02	2.46E-259	4.94E-323
NKX2-5	NKX2-5	5	3.41E-01	TP53TG3D	16	5.81E-10	2.18E-226	6.66E-02	7.61E-02	1.15E-228	0.00E + 00
ASIC2	ASIC2	17	2.24E-02	RPS16P5	6	4.08E-05	3.39E-226	1.12E-01	7.77E-02	1.76E-158	8.01E-237
TMEM132E	TMEM132E	17	8.25E-02	LOC100144602	4	9.50E-51	2.04E-213	1.34E-01	1.30E-01	2.27E-142	1.14E-215
TMEM98	TMEM98	17	6.66E-01	LOC100144602	4	9.28E-51	4.89E-213	8.10E-02	1.13E-01	4.06E-144	6.05E-216
SPACA3	SPACA3	17	7.13E-02	LOC100144602	4	1.41E-50	9.18E-211	9.72E-03	1.21E-02	8.78E-141	4.90E-214
ASIC2	ASIC2	17	2.24E-02	OR5B12	11	3.09E-05	2.89E-205	1.97E-01	1.18E-01	3.63E-141	1.18E-259
CCL1	CCL1	17	6.53E-02	TINF2	14	3.57E-22	3.85E-205	1.23E-01	1.31E-01	1.52E-157	3.33E-210
SCN2A	SCN2A	2	1.41E-01	DEFB4B	8	1.30E-32	5.53E-203	1.88E-02	4.91E-02	1.30E-104	2.67E-236
ZNF254	ZNF254	19	4.41E-01	OR2V1	5	2.20E-13	1.19E-183	2.35E-02	4.91E-02	5.50E-225	2.88E-259
KRT5	KRT5	12	2.45E-02	OR5K1	3	1.03E-28	3.26E-177	1.15E-02	2.33E-02	2.29E-201	5.79E-199
FNDC8	FNDC8	17	4.83E-01	LOC100144602	4	8.13E-49	3.46E-172	4.38E-03	6.19E-02	1.20E-124	8.03E-175
CCT6B	CCT6B	17	3.25E-01	LOC100144602	4	8.81E-49	4.24E-172	7.49E-03	3.59E-02	3.10E-125	1.72E-177
TMEM163	TMEM163	2	1.16E-01	HIST1H4H	6	1.79E-09	8.50E-170	6.74E-02	4.79E-02	3.40E-112	1.19E-25
ASIC2	ASIC2	17	2.24E-02	LOC100144602	4	9.66E-51	6.06E-165	9.22E-01	8.72E-01	3.82E-104	9.50E-236
KRT5	KRT5	12	2.45E-02	IFNA7	9	3.70E-16	2.43E-163	2.26E-02	1.83E-02	2.15E-178	2.14E-211

**Fig. 5:**
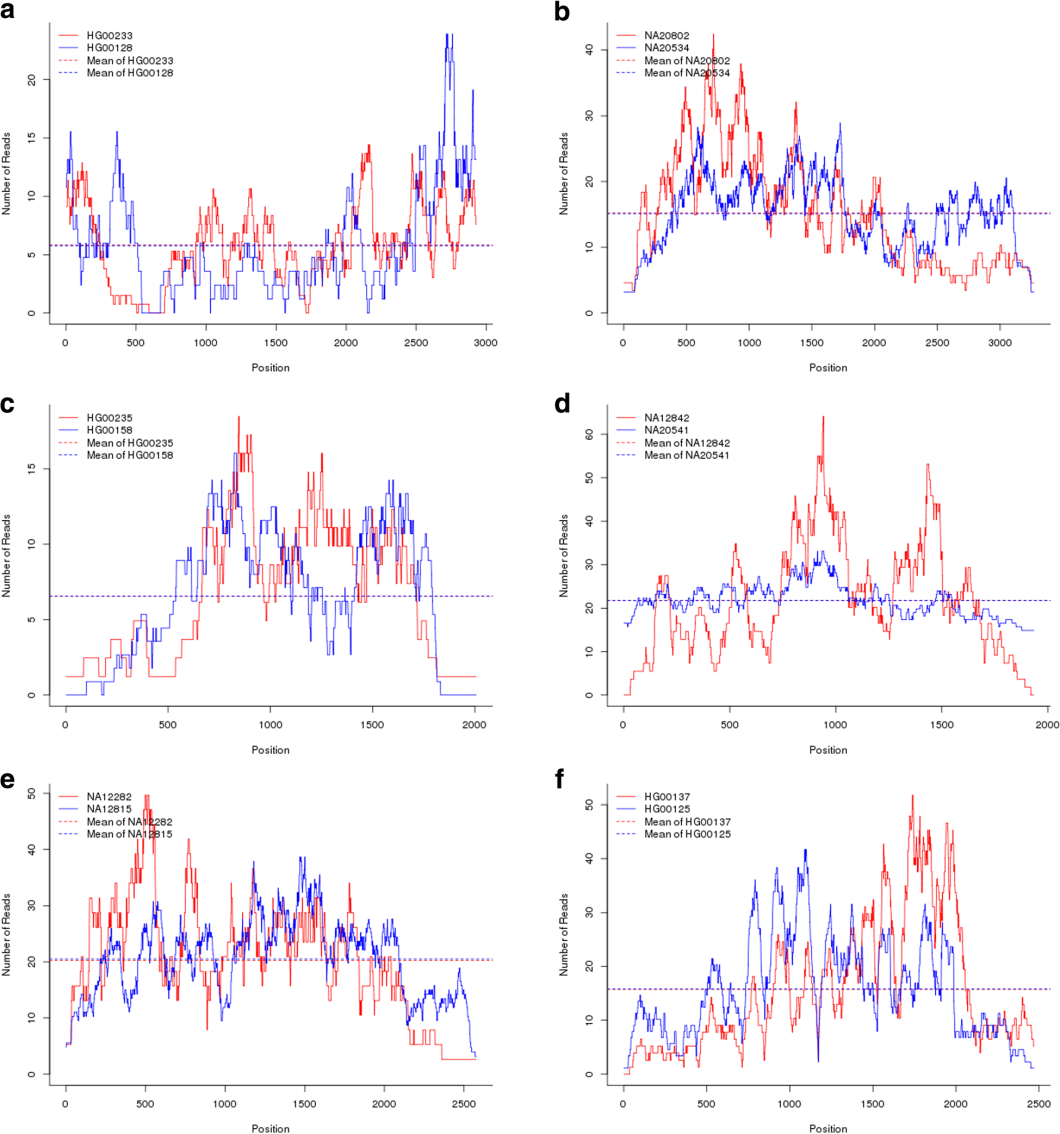
**a**. RNA-seq profile of the gene *PLA2G4A* where the curve represented the number of reads as a function of the genomic position. The dotted line denoted the overall expression of the gene *PLA2G4A.* **b**. RNA-seq profile of the gene *PLA2G6* where the curve represented the number of reads as a function of the genomic position. The dotted line denoted the overall expression of the gene *PLA2G6.* **c**. RNA-seq profile of the gene *PLAUR* where the curve represented the number of reads as a function of the genomic position. The dotted line denoted the overall expression of the gene *PLAUR.* **d**. RNA-seq profile of the gene *PLD4* where the curve represented the number of reads as a function of the genomic position. The dotted line denoted the overall expression of the gene *PLD4.* **e**. RNA-seq profile of the gene *PLD6* where the curve represented the number of reads as a function of genomic position. The dotted line denoted the overall expression of the gene *PLD6.* **f**. RNA-seq profile of the gene *PLEKHA3* where the curve represented the number of reads as a function of the genomic position. The dotted line denoted the overall expression of the gene *PLEKHA3*

To further assess the validity of the BFGM for epistasis analysis with RNA-seq data, we randomly selected six pairs of genes from the significant 162361 gene-gene interactions. The *P*-values for testing the interactions of six pairs of genes using the BFGM and SFGM were summarized in Table 5. Table 5 showed that six significant interactions identified by the BFGM significantly influenced read count variation at least at one genomic position within the gene. To explain why the BFGM had higher power to detect interaction than the SFGM, we presented Fig. 5a-f showing the RNA-seq profiles and overall expression level of the genes *PLA2G4A, PLA2G6, PLAUR, PLD4, PLD6* and *PLEKHA3* of two individuals, respectively. These figures showed that the overall expression levels of the individuals were the same, but their RNA-seq profiles were quite different. This demonstrated that unlike the RNA-seq profiles, the overall expression levels cannot capture the expression variation across the genes. Therefore, the SFGM using summary statistics as a trait will have less power to detect the interaction than the BFGM using the RNA-seq profiles as a function-valued trait.

**Table 5:** The *P*-values of randomly selected 6 pairs of genes from the significant 162361 gene-gene interactions

		*P*-values			
GENE1	GENE2	BFGM	SFGM		
			RPKM	DESeq	RNAmin
PLA2G4A	OR7E2P	1.18E-18	3.68E-02	2.79E-02	1.68E-28
PLA2G6	FGF14-AS2	2.43E-14	8.19E-01	4.90E-01	1.42E-26
PLAUR	CAPNS2	5.14E-13	1.44E-01	9.97E-01	5.15E-22
PLD4	RPL19P12	2.68E-16	4.68E-01	3.49E-01	4.90E-19
PLD6	GHSR	1.07E-11	1.32E-01	4.00E-02	1.48E-28
PLEKHA3	ORMDL2	2.16E-13	4.98E-01	8.43E-01	1.80E-33

To investigate whether the top 20 interactions were caused by the linkage disequilibrium (LD) or not, we listed the maximum of *r* ^2^ between all possible SNPs in the top 20 significantly interacting pairs of genes and the *P*-values for testing their presence of LD in Table 6. We did not observe the strong LD between the interacting genes.

**Table 6:** The maximum of r^2^ between all possible SNPs in top 20 significantly interacting pairs of genes

GENE1	GENE2	*r* ^2^	*P*-value
ULK4	C19orf70	0.00014	0.14974
ULK4	OR10A2	0.00145	0.16383
CCDC13	TMEM121	0.00175	0.19444
ULK4	PSMC5	0.00019	0.11420
ULK4	COX5B	0.00020	0.12871
NKX2-5	TP53TG3D	0.00111	0.18750
ASIC2	RPS16P5	0.00029	0.12406
TMEM132E	LOC100144602	0.00064	0.10577
TMEM98	LOC100144602	0.00043	0.10606
SPACA3	LOC100144602	0.00108	0.09375
ASIC2	OR5B12	0.00048	0.11716
CCL1	TINF2	0.00017	0.13846
SCN2A	DEFB4B	0.00016	0.07823
ZNF254	OR2V1	0.00058	0.14939
KRT5	OR5K1	0.00017	0.03571
FNDC8	LOC100144602	0.00025	0.05882
CCT6B	LOC100144602	0.00029	0.05000
TMEM163	HIST1H4H	0.00193	0.14273
ASIC2	LOC100144602	0.00059	0.12145
KRT5	IFNA7	0.00023	0.07143

### Interactions in the MAPK signaling pathway

**Fig. 6:**
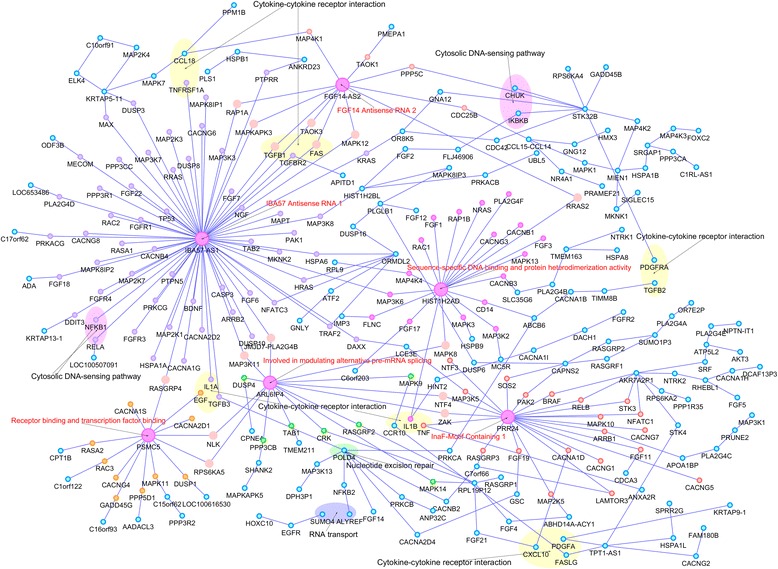
Networks of 317 pairs of genes from 281 genes showing the significant evidence of *cis*-trans interactions as identified by BFGM

To show the detailed interaction structure, we presented the results of 331 significant *cis*-trans interactions in the MAPK signaling pathway in the Additional file 2: Table S3 where min *P*-values indicated that the functional regression model with functional response and discrete predictors was used to test for the interaction for all possible pairs of SNPs within two genes and minimum of *P*-values of the tests was listed in the Additional file 2: Table S3. The column “SNP pair” listed their corresponding pair of SNPs reaching the minimum of the *P*-values and their chromosome locations. From Additional file 2: Table S3 we had several significant observations. First, we observed that the majority of interacting genes were located in different chromosomes, which implied that interactions were not caused by the linkage disequilibrium (LD). Second, we observed that large proportions of interacting genes did not show significant evidence of marginal association. This demonstrated that if we only selected the genes with significant marginal association for epistasis analysis, many interactions would be missed. Third, in general, the function-value-based epistasis analysis (BFGM, min *P*-values) had much smaller *P*-values than the summary statistic-based epistasis analysis (SFGM). Fourth, we observed that the genes interacting with the genes in MAPK signaling pathway were in 147 other pathways, including cytokine-cytokine receptor interaction, Cytosolic DNA-sensing pathway, DNA replicationamong others. Fifth, it was interesting to observe that the interacting genes formed a large connected network with 281 nodes and 317 edges (Fig. 6). We observed hub genes *IBA57-AS1* with 67 connections, *HIST1H2AD* with 21 connections, *PRR24* with 18 connections and *ARL6IP4* with 14 connections. *HIST1H2AD* is a core component of nucleosome and plays a central role in transcription regulation. *ARL6IP4* functions as a splicing inhibitor [25].

### Gene ontology and KEGG pathway enrichment analysis

Gene ontology enrichment analysis was performed on the genes in the identified 162361 pairs of significant *cis*-trans interactions influencing the transcription to discover overrepresented functional biological groupings with interactions. Our analysis was performed using the biological process, cellular component and molecular function categories of the gene ontology.

**Fig. 7:**
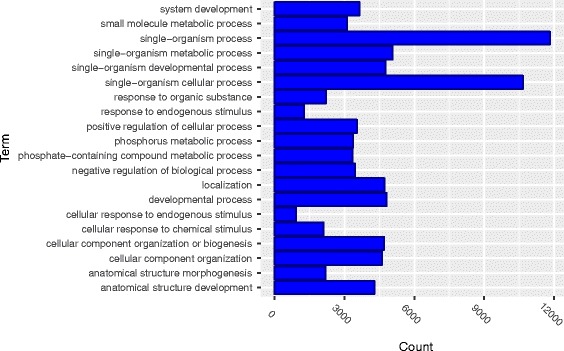
Gene ontology (GO) enrichment analysis of *cis*-trans interactions: enriched in the category of biological processes

**Fig. 8:**
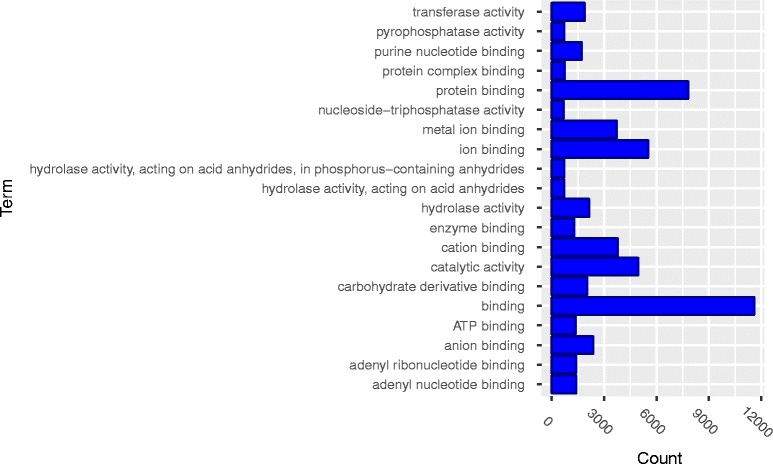
Gene ontology (GO) enrichment analysis of *cis*-trans interactions: enriched in the category of molecular functions

**Fig. 9:**
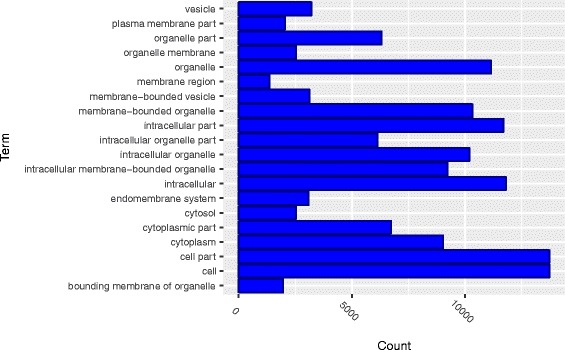
Gene ontology (GO) enrichment analysis of *cis*-trans interactions: enriched in the category of cellular components

Ontology enrichment analysis found that *cis*-trans interactions were significantly enriched in biological processes (BP) including a single organism process, single organism cellular process, single organism metabolic process and development process (Fig. 7) and molecular functions that were primarily related to catalytic and binding activity with *P*-values <10^−4^ (Fig. 8). Ontology enrichment analysis also identified that *cis*-trans interactions were significantly enriched in the cell, intracellular, organelle, and membrane bounded organelle components (Fig. 9).

**Fig. 10:**
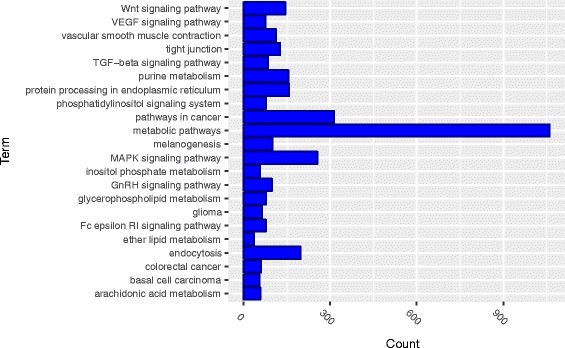
The enrichment analysis of cis-trans interactions in 228 KEGG pathways

The enrichment analysis was also applied to 228 KEGG pathways to identify the pathways that were enriched with *cis*-trans interactions. The results were summarized in Fig. 10. The *cis*-trans interactions were enriched in metabolic pathways, MAPK signaling pathway, pathways in cancer, endocytosis, protein processing in endoplasmic reticulum and Wnt signaling pathway.

## Communities in gene interaction networks

**Fig. 11:**
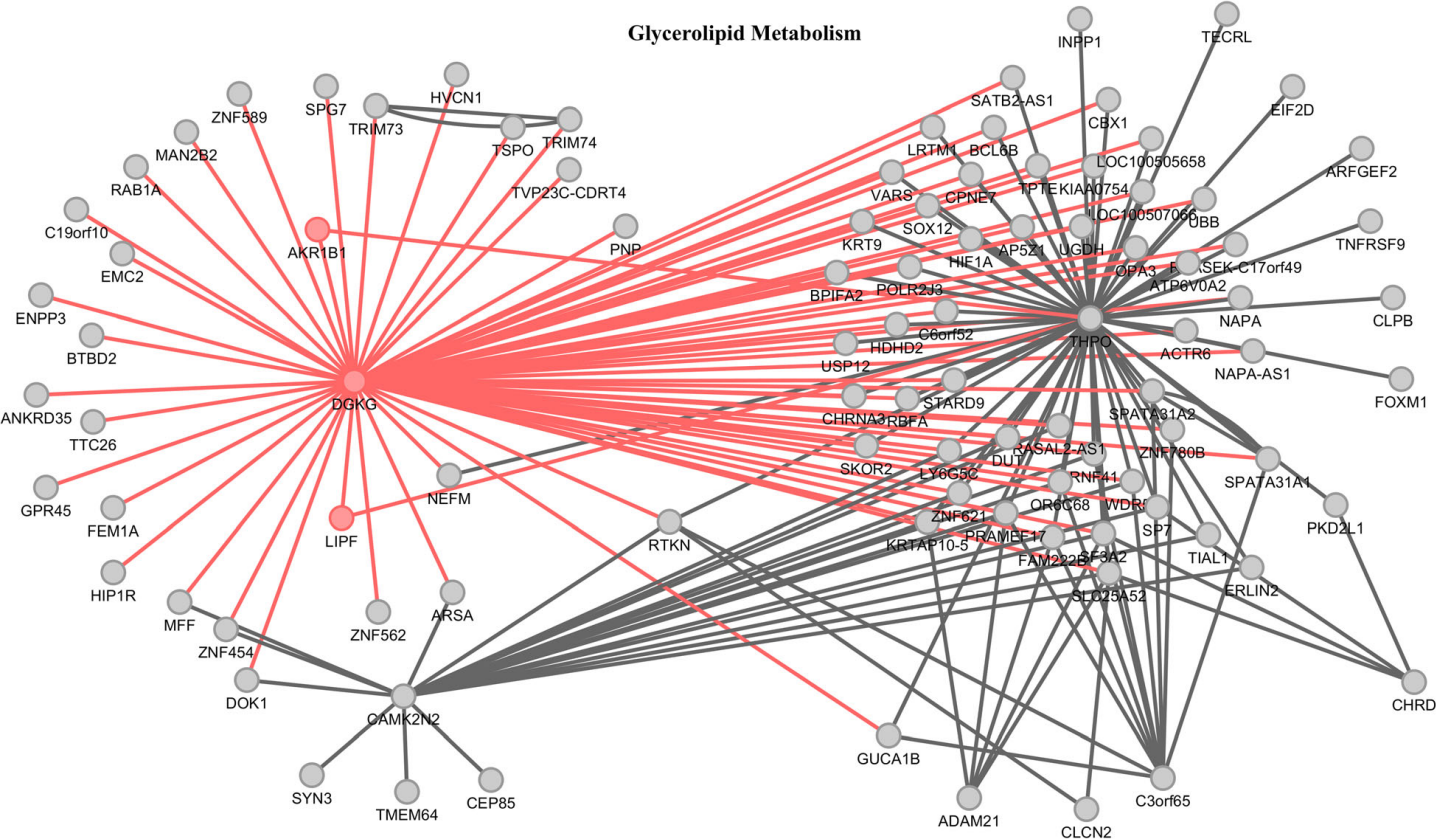
A subnetwork in the 6^*th*^ community with 96 genes and 186 interactions enriched with metabolism where node represents a gene and an edge represents the interaction between the connected gene by the edge

We used random walks in igraph [26] to detect 10 communities from the entire gene-gene interaction network. We used R package GOstats [27] to conduct gene set enrichment analysis. We have identified 29 pathways enriched in 10 communities. Figure 11 showed the 6^*th*^ community with 96 genes and 186 interactions enriched with metabolism (Three of four significantly enriched pathways were metabolism pathways: Glycerolipid metabolism, Nicotinate and nicotinamide metabolism, and Pyrimidine metabolism) where node represents a gene and an edge represents the interaction between the connected gene by the edge. All 10 communities with the enriched pathways (*P*-value < 0.01) are summarized in Additional file 3: Table S4.

## Discussion

In the past, the statistical epistasis of gene expression is defined as variant–variant interactions that regulate gene expression and its analysis has been mainly designed for microarray gene expression data and common variants. Since the dimension of the data for epistasis analysis of gene expression is very high, all the traditional methods for epistasis analysis of gene expression have the limited application to eQTL data. The whole genome epistasis studies of gene expressions have been very limited. The genetic structure of epistasis of gene expressions has not been fully discovered.

The recently developed next-generation mRNA sequencing (RNA-seq) assay generates dozens or even one hundred million short reads of mRNA and WGS also generates millions of SNPs. As a consequence, these genetic variation and gene expression variation data are so densely distributed across the genome that both genetic variation and expression variation can be modeled as a function of genomic location. The RNA-seq profiles can be taken as a function-valued trait. However, the standard multivariate statistical analysis often fails with functional data. The computational burden and correction for multiple tests seriously damage the feasibility of the variant-variant interaction analysis of extremely high dimensional RNA-seq and WGS genotype data. The variant-variant interaction analysis is not suitable for the epistasis analysis of the function-valued traits with NGS data as genotype data. Although the genetic study of quantitative traits has seen wide application and extensive technical development, the quantitative genetic analysis, particularly epistasis analysis of function-valued trait is comparatively less developed. To our knowledge, no statistical methods have been developed for genetic epistasis analysis of function-valued traits with NGS data. In the past few years we have witnessed the rapid development of novel statistical methods for association studies using NGS data. However, these methods might not be appropriate for genetic epistasis analysis of function-valued trait. The quantitative genetic epistasis analysis of rare variants for function-valued traits remains a huge challenge.

The widely used methods for reducing dimensionality of the RNA-seq data use the Poisson distribution, binomial distribution and negative binomial distribution to summarize the RNA-seq profile into a single number to represent the RNA-seq curve. However, these discrete distributions cannot capture the shape and variation of the RNA-seq curve. To illustrate this we presented Additional file 4: Figure S1A showing the real RNA-seq curve, the data simulated by a negative distribution of the gene *LMNB2* and Additional file 4: Figure S1B showing the real RNA-seq curve of the gene *LMNB2* and the curve estimated by the FPCA of the RNA-seq data. We observed that the negative distribution failed to capture the variation of the RNA-seq profile, but the FPCA approximated the RNA-seq curve exceedingly well.

Emergence of the NGS techniques demands a paradigm shift in the analytic methods for eQTL epistasis analysis from standard single-variate or multivariate data analysis to functional data analysis. The BFGM with functional response and functional predictors takes a RNA-seq profile as a functional response and genetic variants across the genomic regions as functional predictors, which can be used to test the association of the entire allelic spectrum of the genetic variation with a function-valued trait and has several remarkable features. First, unlike simple and multiple regressions that discard a large amount of information, the BFGM preserves the intrinsic structure and all the positional-level genetic information. Second, the multiple regressions will not account for the space-ordering of the data and correlation information contained in the data. The BFGM simultaneously employs genetic information of the individual variants and correlation information contained in both RNA-seq and SNP data. Third, both the sign and the size of the heterogeneity will also be incorporated into the test in the BFGM. Fourth, the multicolinearity problem in the BFGM is alleviated. Fifth, the BFGM expands both RNA-seq function and genotype function in terms of orthogonal eigenfunctions, which leads to substantial dimension reduction. The BFGM for genetic epistasis analysis of a function-valued trait which captures key information in the data is expected to open a new route for genetic epistasis analysis of RNA-seq and NGS genotype data.

## Conclusions

We developed a novel functional regression model with both functional response and functional predictors for detection of epistasis influencing RNA-seq variations in humans, which is referred to as the BFGM. The BFGM takes genes as a basic unit of epistasis analysis and utilizes all information contained in both the RNA-seq and SNP data. By large simulations and real data analysis we demonstrated the merits and limitations of the proposed new paradigm of epistasis analysis for the RNA-seq and WGS data.

The new approach uses all genetic information in the genome regions and expression variation information in the target gene to collectively test the interaction between multiple SNPs within the regions influencing the RNA-seq curves. Therefore, the BFGM for interaction analysis overcomes limitations inherent in pair-wise interaction tests with the summary expression level as a scalar trait. By large simulations and real data analysis, we showed that the proposed BFGM substantially increased the power, dramatically reduced the computational burden and substantially outperformed the traditional variant-variant epistasis analysis of summary statistic measured quantitative traits. In real data analysis, we also clearly demonstrate that pairs of SNPs between two genes jointly have significant interaction effects, but individually each pair of SNPs makes a mild contribution to interaction effects.

The previous interaction analyses have mainly focused on the interactions between common and common variants. The distribution of the common and rare variants causing interactions is unknown. Very few genome-wide interaction analyses with the RNA-seq and WGS data, and very few results of significant interaction between rare and rare variants, and rare and common variants have been reported. We analyzed 350 samples of European origin with both RNA-seq and whole genome sequencing data available. We observed the large proportions of pair-wise interactions between rare and rare variants, and rare and common variants. The significant pair-wise interactions between common and common variants were less observed. The results showed that the number of significant *cis*-trans interactions identified by the SFGM with RPKM as overall gene expression level only accounted for 0.16% of the significant *cis*-trans interactions identified by the BFGM with RNA-seq and NGS genotype data. The majority of epistasis analysis for gene expressions used the microarray to measure gene expressions and test interactions for only common variants. Even though the RNA-seq data are available they still converted variation rich RNA-seq data into a single number such as RPKM or other summary statistics. Then, the variant-variant epistasis analysis is conducted on these converted data. That explains why these researches question the universe presence of significant gene-gene interaction influencing gene expressions.

Some researchers suggest that in genome-wide interaction analysis only genes with large or mildly marginal genetic effects should be tested for interaction. However, we observed that the majority of the significantly interacting genes showed no marginal association. These results clearly demonstrated that if we tested interactions for only genes with marginal associations, then many true interactions will be missing.

We are unsure whether interaction is most often presented in isolation, or interacting genes form networks. We identified a large number of *cis*-trans interactions and observed that the interacting genes formed large connected networks with hub genes presented. We found that some hub genes, for example histone modification genes, can globally regulate gene expressions. Enrichment analysis also showed that metabolic pathways, MAPK signaling pathway, pathways in cancer, endocytosis, protein processing in endoplasmic reticulum and Wnt signaling pathway among others were enriched with *cis-*trans interactions.

The results in this paper are preliminary. The confounding factors that cause spurious interactions have not been investigated. The statistical methods for epistasis analysis which remove confounding factors have not been developed. The complete genome-wide epistasis analysis including all *cis* and trans interactions have not been performed. The purpose of this paper is to stimulate further discussions regarding the great challenges we are facing in the epistasis analysis of high dimensional RNA-seq and WGS data.

## Methods

### Functional regression with both functional response and functional predictor models for epistasis analysis

For the convenience of discussion, position level read counts are taken as the RNA-seq profile and is referred to as a function-valued trait. Let *y*_*i*_(*τ*), *τ* ∈ *Τ*_*τ*_ = [0, *Τ*_*τ*_] be the read counts of the *i* ^th^ individual at the genomic position *τ*. Consider two genomic regions (or genes) [*a*_1_, *b*_1_] and [*a*_2_, *b*_2_]. Let *x*_*i*_(*t*) and z_*i*_(*s*) be genotypic functions of the *i* ^th^ individual defined in the regions [*a*_1_, *b*_1_] and [*a*_2_, *b*_2_], respectively. Let *t* and *s* be a genomic position in the first and second genomic regions, respectively. The genotype functions *x*_*i*_(*t*) and z_*i*_(*s*) are defined as

$$ {\mathrm{x}}_i\left(\mathrm{t}\right)=\left\{\begin{array}{c}\hfill 2{P}_m\left(\mathrm{t}\right), \kern2.25em \mathrm{MM}\hfill \\ {}\hfill {P}_m\left(\mathrm{t}\right)\hbox{-} {\mathrm{P}}_{\mathrm{M}}\left(\mathrm{t}\right),\kern0.5em \mathrm{Mm}\hfill \\ {}\hfill -2{P}_M\left(\mathrm{t}\right), \kern1.5em \mathrm{mm}\hfill \end{array}\right.,\kern2em {\mathrm{z}}_i\left(\mathrm{s}\right)=\left\{\begin{array}{c}\hfill 2{P}_m\left(\mathrm{s}\right), \kern2.25em \mathrm{MM}\hfill \\ {}\hfill {P}_m\left(\mathrm{s}\right)\kern0.5em \hbox{-} \kern0.5em {\mathrm{P}}_{\mathrm{M}}\left(\mathrm{s}\right),\kern0.5em \mathrm{Mm},\hfill \\ {}\hfill -2{P}_M\left(\mathrm{s}\right), \kern1.5em \mathrm{mm}\hfill \end{array}\right. $$

where M and m are two alleles of the marker at the genomic position *t* and *s*, *P*_*M*_(*t*) and *P*_*m*_(*t*), and *P*_M_(*s*), *P*_*m*_(*s*) are the frequencies of the alleles M and m at the genomic positions *t* and *s*, respectively. Consider a functional regression model with functional response and functional predictors (BFGM):

1 $$ {y}_i\left(\tau \right)=\mu \left(\tau \right)+{W}_i^T\omega \left(\tau \right)+{\displaystyle {\int}_T{x}_i(t)\alpha \left( t,\tau \right) dt+{\displaystyle {\int}_S{z}_i(s)\beta \left( s,\tau \right) ds+{\displaystyle {\int}_T{\displaystyle {\int}_S{x}_i(t){z}_i(s)\gamma \left( t, s,\tau \right) ds dt}}}}+{\varepsilon}_i\left(\tau \right) $$

where *μ*(*τ*) is an overall mean function at the genomic position *τ*, *W*_*i*_ is a vector of covariates for *i* ^*th*^ individual, *ω*(*τ*) is a vector of effects associated with the covariates, *α*(*t*, *τ*) is a genetic additive effect function at genomic position *t* of the first gene and genomic position *τ* of the RNA-seq profile, *β*(*s*, *τ*) is a genetic additive effect function at genomic positions *s* of the second gene and the genomic position *τ*, *γ*(*t*, *s*, *τ*) is an interaction effect function between two putative quantitative trait loci (QTLs) located at the genomic positions *t* and *s* influencing the read counts at the genomic position *τ*, and *ε*_*i*_(*τ*) is a residual function of the unexplained effect for the *i* ^*th*^ individual at the genomic position *τ*. The interaction function is measured by double integrals of the genotype function in two genes.

### Estimation of interaction effect function

We assume that both position level read count function and genotype functions are centered. The genotype functions *x*_*i*_(*t*) and *z*_*i*_(*s*) are expanded in terms of the orthonormal basis function as:

2 $$ {x}_i(t)={\displaystyle \sum_{j=1}^{\infty }{\xi}_{i j}{\phi}_j(t)}\kern1em \mathrm{and}\kern1em {z}_i(s)={\displaystyle \sum_{l=1}^{\infty }{\eta}_{{}_{i l}}{\psi}_{{}_l}(s),} $$

where ${\phi}_{{}_j}(t)$ and *ψ*_*l*_(*s*) are sequences of the orthonormal basis functions. The expansion coefficients *ξ*_*ij*_ and *η*_*il*_ are estimated by

3 $$ {\xi}_{i j}={\displaystyle \underset{T}{\int }{x}_{{}_i}(t){\phi}_{{}_j}(t) dt\kern0.75em }\kern0.5em \mathrm{and}\kern1.5em {\eta}_{{}_{i l}}={\displaystyle \underset{S}{\int }{z}_{{}_i}(s){\psi}_{{}_l}(s) ds} $$

In practice, numerical methods for the integral will be used to calculate the expansion coefficients. Substituting Eq. (2) into Eq. (1), we obtain

4 $$ {y}_i\left(\tau \right)=\mu \left(\tau \right)+{W}_i^T\omega \left(\tau \right)+{\displaystyle {\sum}_{j=1}^J{\xi}_{i j}}{\alpha}_j\left(\tau \right)+{\displaystyle {\sum}_{l=1}^L{\eta}_{i l}}{\beta}_l\left(\tau \right)+{\displaystyle {\sum}_{j=1}^J{\displaystyle {\sum}_{l=1}^L{\xi}_{i j}}}{\eta}_{i l}{\gamma}_{j l}\left(\tau \right)+{\varepsilon}_i\left(\tau \right), $$

where

$$ \begin{array}{l}{\alpha}_{{}_j}\left(\tau \right)={\displaystyle {\int}_T\alpha \left( t,\tau \right){\phi}_{{}_j}(t) dt,}\kern0.5em {\beta}_{{}_l}\left(\tau \right)={\displaystyle {\int}_S\beta \left( s,\tau \right){\psi}_l(s) ds}\kern0.5em \mathrm{and}\\ {}\kern0.5em {\gamma}_{{}_{j l}}\left(\tau \right)={\displaystyle {\int}_T{\displaystyle {\int}_S\gamma \left( t, s,\tau \right){\phi}_{{}_j}(t){\psi}_{{}_l}(s) dt ds}}.\end{array} $$

The parameters *α*_*j*_(*τ*), *β*_*l*_(*τ*) and *γ*_*jl*_(*τ*) are referred to as genetic additive effect and additive x additive effect score functions. These score functions can also be viewed as the expansion coefficients of the genetic effect functions with respect to orthonormal basis functions:

$$ \alpha \left( t,\tau \right)={\displaystyle \sum_j{\alpha}_j\left(\tau \right){\phi}_j(t),\beta \left( s,\tau \right)={\displaystyle \sum_l{\beta}_l\left(\tau \right){\psi}_l(s)\ \mathrm{and}\ \gamma \left( s, t\right)={\displaystyle \sum_j{\displaystyle \sum_l{\gamma}_{j l}\left(\tau \right){\phi}_j(s){\psi}_l(t)}}}}. $$

Equation (4) can be written in a vector form:

5 $$ Y\left(\tau \right)=\mathrm{E}\mu \left(\tau \right)+ W\omega \left(\tau \right)+\xi \alpha \left(\tau \right)+\eta \beta \left(\tau \right)+\varGamma \gamma \left(\tau \right)+\varepsilon \left(\tau \right)\kern0.5em , $$

where *Y*(*τ*), *μ*(*τ*), *ω*(*τ*), *α*(*τ*), *β*(*τ*) and *γ*(*τ*) are vectors, *W*, *ξ*, *η* and *Γ* are matrices.

Expanding *Y*(*τ*), *μ*(*τ*), *ω*(*τ*), *α*(*τ*), *β*(*τ*), *γ*(*τ*) and *ε*(*τ*) in terms of the orthogonal basis functions yield

$$ \begin{array}{l}{y}_{{}_i}\left(\tau \right)={\displaystyle {\sum}_{k=1}^K{y}_{{}_{i k}}{\theta}_{{}_k}\left(\tau \right)}\kern0.5em ,\kern0.5em \mu \left(\tau \right)={\displaystyle {\sum}_{k=1}^K{\mu}_{{}_k}{\theta}_{{}_k}\left(\tau \right)}\kern0.5em ,\kern0.5em {\omega}_{{}_j}\left(\tau \right)={\displaystyle {\sum}_{k=1}^K{\omega}_{{}_{j k}}{\theta}_{{}_k}\left(\tau \right)}\kern0.5em ,\\ {}\kern1em {\alpha}_{{}_j}\left(\tau \right)={\displaystyle {\sum}_{k=1}^K{\alpha}_{{}_{j k}}{\theta}_{{}_k}\left(\tau \right)}\kern0.5em ,\kern0.5em {\beta}_{{}_j}\left(\tau \right)={\displaystyle {\sum}_{k=1}^K{\beta}_{{}_{j k}}{\theta}_{{}_k}\left(\tau \right)}\kern0.5em ,\kern0.5em {\gamma}_{{}_{j l}}\left(\tau \right)={\displaystyle {\sum}_{k=1}^K{\gamma}_{{}_{j l k}}{\theta}_{{}_k}\left(\tau \right)}\kern0.5em ,\kern1em \mathrm{and}\\ {}\kern1em {\varepsilon}_{{}_i}\left(\tau \right)={\displaystyle {\sum}_{k=1}^K{\varepsilon}_{{}_{i k}}{\theta}_{{}_k}\left(\tau \right)}.\end{array} $$

Define expansion coefficient vectors and matrices as follows.

$$ \begin{array}{l} Y=\left[\begin{array}{ccc}\hfill {y}_{{}_{11}}\hfill & \hfill \cdots \hfill & \hfill {y}_{{}_{1 K}}\hfill \\ {}\hfill \vdots \hfill & \hfill \ddots \hfill & \hfill \vdots \hfill \\ {}\hfill {y}_{{}_{n1}}\hfill & \hfill \cdots \hfill & \hfill {y}_{{}_{n K}}\hfill \end{array}\right],\kern0.5em \mu ={\left[\begin{array}{c}\hfill {\mu}_{{}_1}\hfill \\ {}\hfill \vdots \hfill \\ {}\hfill {\mu}_{{}_K}\hfill \end{array}\right]}^{\mathrm{T}},\kern0.5em  E=\left[\begin{array}{c}\hfill 1\hfill \\ {}\hfill \vdots \hfill \\ {}\hfill 1\hfill \end{array}\right],\kern1em \omega =\left[\begin{array}{ccc}\hfill {\omega}_{{}_{11}}\hfill & \hfill \cdots \hfill & \hfill {\omega}_{{}_{1 K}}\hfill \\ {}\hfill \vdots \hfill & \hfill \ddots \hfill & \hfill \vdots \hfill \\ {}\hfill {\omega}_{{}_{d1}}\hfill & \hfill \cdots \hfill & \hfill {\omega}_{{}_{d K}}\hfill \end{array}\right],\kern0.5em \\ {}\alpha =\left[\begin{array}{ccc}\hfill {\alpha}_{{}_{11}}\hfill & \hfill \cdots \hfill & \hfill {\alpha}_{{}_{1 K}}\hfill \\ {}\hfill \vdots \hfill & \hfill \ddots \hfill & \hfill \vdots \hfill \\ {}\hfill {\alpha}_{{}_{J1}}\hfill & \hfill \cdots \hfill & \hfill {\alpha}_{{}_{J K}}\hfill \end{array}\right],\end{array} $$

$$ \beta =\left[\begin{array}{ccc}\hfill {\beta}_{{}_{11}}\hfill & \hfill \cdots \hfill & \hfill {\beta}_{{}_{1 K}}\hfill \\ {}\hfill \vdots \hfill & \hfill \ddots \hfill & \hfill \vdots \hfill \\ {}\hfill {\beta}_{{}_{L1}}\hfill & \hfill \cdots \hfill & \hfill {\beta}_{{}_{L K}}\hfill \end{array}\right],\kern1em \gamma =\left[\begin{array}{ccc}\hfill {\gamma}_{{}_{111}}\hfill & \hfill \cdots \hfill & \hfill {\gamma}_{{}_{11 K}}\hfill \\ {}\hfill \vdots \hfill & \hfill \ddots \hfill & \hfill \vdots \hfill \\ {}\hfill {\gamma}_{{}_{JL1}}\hfill & \hfill \cdots \hfill & \hfill {\gamma}_{{}_{JL K}}\hfill \end{array}\right]\kern1em \mathrm{and}\kern0.5em \varepsilon =\left[\begin{array}{ccc}\hfill {\varepsilon}_{{}_{11}}\hfill & \hfill \cdots \hfill & \hfill {\varepsilon}_{{}_{1 K}}\hfill \\ {}\hfill \vdots \hfill & \hfill \ddots \hfill & \hfill \vdots \hfill \\ {}\hfill {\varepsilon}_{{}_{n1}}\hfill & \hfill \cdots \hfill & \hfill {\varepsilon}_{{}_{n K}}\hfill \end{array}\right]. $$

Thus, substituting the above expansion into Eq. (5) gives

6 $$ Y\theta \left(\tau \right)=\mu \theta \left(\tau \right)+ W\omega \theta \left(\tau \right)+\xi \alpha \theta \left(\tau \right)+\eta \beta \theta \left(\tau \right)+\varGamma \gamma \theta \left(\tau \right)+\varepsilon \theta \left(\tau \right), $$

where

$$ \begin{array}{c} W=\left[\begin{array}{ccc}\hfill {W}_{{}_{11}}\hfill & \hfill \cdots \hfill & \hfill {W}_{{}_{1 d}}\hfill \\ {}\hfill \vdots \hfill & \hfill \ddots \hfill & \hfill \vdots \hfill \\ {}\hfill {W}_{{}_{n1}}\hfill & \hfill \cdots \hfill & \hfill {W}_{{}_{n d}}\hfill \end{array}\right],\kern1.5em \xi =\left[\begin{array}{ccc}\hfill {\xi}_{{}_{11}}\hfill & \hfill \cdots \hfill & \hfill {\xi}_{{}_{1 J}}\hfill \\ {}\hfill \vdots \hfill & \hfill \ddots \hfill & \hfill \vdots \hfill \\ {}\hfill {\xi}_{{}_{n1}}\hfill & \hfill \cdots \hfill & \hfill {\xi}_{{}_{n J}}\hfill \end{array}\right],\kern1em \eta =\left[\begin{array}{ccc}\hfill {\eta}_{{}_{11}}\hfill & \hfill \cdots \hfill & \hfill {\eta}_{{}_{1 L}}\hfill \\ {}\hfill \vdots \hfill & \hfill \ddots \hfill & \hfill \vdots \hfill \\ {}\hfill {\eta}_{{}_{n1}}\hfill & \hfill \cdots \hfill & \hfill {\eta}_{{}_{n L}}\hfill \end{array}\right]\kern1em \mathrm{and}\\ {}\\ {}\varGamma =\left[\begin{array}{c}\hfill {\upxi}_1^T\otimes {\upeta}_1^T\hfill \\ {}\hfill \vdots \hfill \\ {}\hfill {\upxi}_n^T\otimes {\upeta}_n^T\hfill \end{array}\right]=\left[\begin{array}{ccc}\hfill \begin{array}{ccc}\hfill {\upxi}_{{}_{11}}{\upeta}_{{}_{11}}\hfill & \hfill \cdots \hfill & \hfill {\upxi}_{{}_{11}}{\upeta}_{{}_{1 L}}\hfill \end{array}\hfill & \hfill \cdots \hfill & \hfill \begin{array}{ccc}\hfill {\upxi}_{{}_{1 J}}{\upeta}_{{}_{11}}\hfill & \hfill \cdots \hfill & \hfill {\upxi}_{{}_{1 J}}{\upeta}_{{}_{1 L}}\hfill \end{array}\hfill \\ {}\hfill \begin{array}{ccc}\hfill \cdots \hfill & \hfill \cdots \hfill & \hfill \cdots \hfill \end{array}\hfill & \hfill \cdots \hfill & \hfill \begin{array}{ccc}\hfill \cdots \hfill & \hfill \cdots \hfill & \hfill \cdots \hfill \end{array}\hfill \\ {}\hfill \begin{array}{ccc}\hfill {\upxi}_{{}_{n1}}{\upeta}_{{}_{n1}}\hfill & \hfill \cdots \hfill & \hfill {\upxi}_{{}_{n1}}{\upeta}_{{}_{n L}}\hfill \end{array}\hfill & \hfill \cdots \hfill & \hfill \begin{array}{ccc}\hfill {\upxi}_{{}_{n J}}{\upeta}_{{}_{n1}}\hfill & \hfill \cdots \hfill & \hfill {\upxi}_{{}_{n J}}{\upeta}_{{}_{n L}}\hfill \end{array}\hfill \end{array}\right].\end{array} $$

Since Eq. (6) holds for every genomic position *τ*, the coefficients on both sides of Eq. (6) should be equal. Therefore, the functional regression model (6) can be further transformed to standard multivariate multiple regression:

7 $$ Y= E\mu + W\omega +\xi \alpha +\eta \beta +\varGamma \gamma +\varepsilon . $$

Let

$$ A=\left[\begin{array}{cccc}\hfill \begin{array}{cc}\hfill E\hfill & \hfill W\hfill \end{array}\hfill & \hfill \xi \hfill & \hfill \eta \hfill & \hfill \varGamma \hfill \end{array}\right]\kern0.5em \mathrm{and}\kern0.5em  b=\left[\begin{array}{c}\hfill \begin{array}{c}\hfill \mu \hfill \\ {}\hfill \omega \hfill \end{array}\hfill \\ {}\hfill \alpha \hfill \\ {}\hfill \beta \hfill \\ {}\hfill \gamma \hfill \end{array}\right]. $$

Equation (7) can be rewritten as

8 $$ Y= Ab+\varepsilon . $$

The standard least square estimators of *b* is

9 $$ \widehat{b}={\left({A}^T A\right)}^{-1}{A}^T Y. $$

The covariance matrix *Σ* is estimated by

10 $$ \widehat{\varSigma}=\frac{{\left( Y- A\widehat{b}\right)}^T\left( Y- A\widehat{b}\right)}{\left( n-\left(1+ d+ J+ L+ J L\right)\right)\mathrm{K}}. $$

### Test statistic

An essential problem in genetic epistasis analysis of the function-valued traits is to test the interaction between two genomic regions (or genes). Formally, we investigate the problem of testing the following hypothesis:

$$ \gamma \left( t, s,\tau \right)=0,\forall t\in \left[{a}_1,{b}_1\right], s\in \left[{a}_2,{b}_2\right],\tau \in \left[0,{T}_{\tau}\right], $$

which is equivalent to testing the hypothesis:

11 $$ {H}_0:\gamma =0. $$

Let *vec* denote the vector operation. To develop test statistics, we begin with calculating the covariance matrix of the $v e c\left(\widehat{b}\right)$. We assume that

12 $$ \operatorname{var}\left( vec\left(\varepsilon \right)\right)=\varSigma \otimes {I}_n. $$

Recall that

$$ v e c\left(\widehat{b}\right)=\left[{I}_K\otimes {\left({A}^T A\right)}^{-1}{A}^T\right] v e c(Y). $$

Therefore, we have

13 $$ \begin{array}{l}\operatorname{var}\left( vec\left(\widehat{b}\right)\right)=\left[{I}_K\otimes {\left({A}^T A\right)}^{-1}{A}^T\right]\left(\varSigma \otimes {I}_n\right)\left[{I}_K\otimes A{\left({A}^T A\right)}^{-1}\right]\\ {}\kern7.5em =\varSigma \otimes {\left({A}^T A\right)}^{-1}\end{array} $$

Let *Λ* be a matrix consisting of the last *JLK* columns and *JLK* rows of the covariance matrix $\operatorname{var}\left( vec\left(\widehat{b}\right)\right)$ and $\widehat{\gamma}$ be the estimators of interaction which can be obtained by extracting the last *JL* rows of the estimators of the matrix $\widehat{b}$. Define the test statistic for testing the interaction between two genomic regions [*a*_1_, *b*_1_] and [*a*_2_, *b*_2_] as

14 $$ {T}_I= v e c{\left(\widehat{\gamma}\right)}^T{\varLambda}^{-1} v e c\left(\widehat{\gamma}\right). $$

Then, under the null hypothesis *H*_0_ : *γ* = 0, *T*_*I*_ is asymptotically distributed as a central *χ*_(*JLK*)_^2^ with degrees of freedom *JLK* or the rank of the matrix *Λ*.

### Null distribution of test statistics

To examine the null distribution of test statistics, we performed a series of simulation studies to compare their empirical levels with the nominal ones. We calculated the type I error under three models. We first assumed the model with no marginal effects:

***Model 1*** (no marginal effect):

15 $$ {y}_i\left(\tau \right)=\mu \left(\tau \right)+{\varepsilon}_i\left(\tau \right), $$

where *μ*(*τ*) is the overall mean at the genomic position *τ*, *y*_*i*_(*τ*) is the normalized number of reads at the genomic position *τ* of the *i* ^*th*^ individual and *ε*_*i*_(*τ*) is an error stochastic process. The errors should be correlated stochastic process. The theoretic models for the errors are unclear. They were estimated from the data. The procedures for generating mean *μ*(*τ*) and errors *ε*_*i*_(*τ*) consisted of the following steps.

Step 1: We randomly sampled 100 genes from the whole real RNA-seq dataset. Let *k* index genes, *j* index the genomic positions and *i* index the samples. Assume that the gene *k* is located in the interval [*a*_*k*_, *b*_*k*_]. Let *x*_*ikj*_ , (*i* = 1, …, *n*, *k* = 1, …, 100, *j* = 1, …, *s*_*k*_) be the observed count of reads of the gene *k* in the genomic position *j* of the *i* ^*th*^ individual where the length of gene *k* is denoted *s*_*k*_. For each genomic position, we define an *n* dimensional vector:

*x*_*kj*_ = [*x*_1*kj*_, …, *x*_*nkj*_]^*T*^.

Step 2. Let *m* be the median length of 100 genes. In our dataset, *m* = 2, 456.

Step 3. Re-map the original RNA-seq data of 100 genes to the interval [0, 1] using transformation $\frac{j-{a}_k}{b_k-{a}_k}$. Then, estimate the count of reads on position $0,\frac{1}{m},\frac{2}{m},\dots, 1$ from the original RNA-seq data of the 100 genes using local polynomial regression (LOESS). The estimated count of reads of the gene *k* in the genomic position *j* of the *i* ^*th*^ individual at the equally distributed new positions $0,\frac{1}{m},\frac{2}{m},\dots, 1$ are denoted by *y*_*ikj*_. Define vector

$$ {y}_{kj}={\left[{y}_{1 kj},\dots, {y}_{nkj}\right]}^T, k=1,\dots, 100, j=1,\dots, m. $$

Step 4. Compute the means of the re-mapped the RNA-seq data over 100 genes and over *n* samples: ${\overline{y}}_{ij}=\frac{1}{100}{\displaystyle {\sum}_{k=1}^{100}{y}_{ikj}}, i=1,\dots, n, j=1,\dots, m$ and ${\overline{y}}_j=\frac{1}{100\times n}{\displaystyle {\sum}_{i=1}^n{\displaystyle {\sum}_{k=1}^{100}{y}_{i kj}}}, j=1,\dots, m$.

Define the mean vector of the re-mapped counts of reads: $\overline{y}={\left[{\overline{y}}_1,\dots, {\overline{y}}_m\right]}^T$.

Step 5. Compute the mean function *μ*(*τ*). Pooling all the re-mapped data:

$$ Y=\left[\begin{array}{ccc}\hfill {\overline{y}}_{11}\hfill & \hfill \cdots \hfill & \hfill {\overline{y}}_{1 m}\hfill \\ {}\hfill \vdots \hfill & \hfill \vdots \hfill & \hfill \vdots \hfill \\ {}\hfill {\overline{y}}_{n1}\hfill & \hfill \vdots \hfill & \hfill {\overline{y}}_{n m}\hfill \end{array}\right]. $$

Use the pooled data to perform FPCA, which leads to functional principal component expansion:

$$ {y}_i\left(\tau \right)={\displaystyle {\sum}_{l=1}^L{\xi}_{{}_{i l}}{\beta}_{{}_l}\left(\tau \right)}, i=1,\dots, n, $$

where *β*_*l*_(*τ*) are the functional principal components.

Calculate ${\overline{\xi}}_l=\frac{1}{n}{\displaystyle {\sum}_{i=1}^n{\xi}_{i l}}, l=1,\dots, L$. Using the averaged functional principal component score, we compute the mean *μ*(*τ*) as follows:

$$ \mu \left(\tau \right)={\displaystyle {\sum}_{l=1}^L{\overline{\xi}}_l}{\beta}_l\left(\tau \right). $$

Step 6. Define the centralized RNA-seq data matrix:

$$ Z=\left[\begin{array}{ccc}\hfill {z}_{11}\hfill & \hfill \cdots \hfill & \hfill {z}_{1 m}\hfill \\ {}\hfill \vdots \hfill & \hfill \vdots \hfill & \hfill \vdots \hfill \\ {}\hfill {z}_{n1}\hfill & \hfill \cdots \hfill & \hfill {z}_{n m}\hfill \end{array}\right], $$

where $$ {Z}_{ij}={\overline{y}}_{ij}-{\overline{y}}_j, i=1,\dots, n, j=1,\dots, m $$.

We perform FPCA on the centralized dataset *Z* where means of the RNA-seq data at each genomic position over 100 genes is removed and obtain a set of functional principal components (eigenfunctions) {*φ*_1_(*τ*), …, *φ*_*T*_(*τ*)} and functional principal component scores *η*_*it*_, *i* = 1, …, *n*, *t* = 1, …, *T*. Define *T* random variables *η* = [*η*_1_, …, *η*_*T*_] with vectors of their sampling values:*η*_*t*_ = [*η*_1*t*_, …, *η*_*nt*_]^*T*^, *t* = 1, …, *T*.

We then calculate the sampling covariance matrix $\widehat{\varSigma}=\operatorname{cov}\left(\eta, \eta \right)$.Assume that the scores of the residuals follow a multivariate normal distribution $N\left(0,\widehat{\varSigma}\right)$. Using the normal random variables to generate an *n* sample of vectors *ε*_*i*_ = [*ε*_*i*1_, …, *ε*_*iT*_]. The residuals *ε*_*i*_(*τ*) will be defined as

$$ {\varepsilon}_i\left(\tau \right)={\displaystyle {\sum}_{t=1}^T{\varepsilon}_{i t}{\phi}_t\left(\tau \right), i=1,\dots, n}. $$

***Model 2*** (a marginal effect at the first gene):

16 $$ {y}_i\left(\tau \right)=\mu \left(\tau \right)+{\displaystyle {\sum}_{j=1}^J{x}_{i j}{\alpha}_j\left(\tau \right)+{\varepsilon}_i\left(\tau \right)}, $$

where *μ*(*τ*) is the overall mean at the genomic position *τ*, *ε*_*i*_(*τ*) is an error stochastic process, *x*_*ij*_ is an indicator variable for the genotype of *i* ^*th*^ individual at the *j* ^*th*^ SNP of the first gene, *y*_*i*_(*τ*) is defined as that in model 1. The coefficient *α*_*j*_(*τ*) = *r*_*j*_ ⋅ *α*(*τ*), where *r*_*j*_ is randomly selected from 0.5 to 1.5, is the additive effect function of the *j* ^*th*^ SNP of the first gene, *μ*(*τ*) is obtained by randomly sampling 100 genes from the real RNA-seq and WGS genotype data without interactions and *α*(*τ*) is obtained by randomly sampling 100 genes from the real RNA-seq and WGS genotype data under the condition that one gene have significant main effect, the other gene do not have significant main effect, and these gene pairs are not in the list of significantly interacted gene pairs in our results. The overall mean *μ*(*τ*), effect function *α*(*τ*) and the residuals *ε*_*i*_(*τ*) were similarly simulated as that in Model 1.

***Model 3*** (marginal effects at both the first and the second genes):

17 $$ {y}_i\left(\tau \right)=\mu (t)+{\displaystyle {\sum}_{j=1}^J{x}_{i j}{\alpha}_j\left(\tau \right)+{\displaystyle {\sum}_{k=1}^K{z}_{i k}{\beta}_k\left(\tau \right)+}{\varepsilon}_i\left(\tau \right)}, $$

where *z*_*ik*_ is an indicator variable for the genotype of *i* ^*th*^ individual at the *k* ^*th*^ SNP of the second gene. The genetic additive effect function *β*_*k*_(*τ*_*l*_) is assumed to be equal to *β*_*k*_(*τ*) = *s*_*k*_ *β*(*τ*), where *s*_*k*_ is randomly selected from 0.5 to 1.5, other parameters are defined in Model 2. A total of 100 pairs of genes were randomly selected under the condition that both genes have significant main effect and these gene pairs are not in the list of significant interacted gene pairs in our results. The overall mean function *μ*(*τ*), main effect functions *α*(*τ*) and *β*(*τ*), and residual term *ε*_*i*_(*τ*) were similarly generated as that in Models 1 and 2.

### Power evaluation

To evaluate the performance of the functional regression model for testing epistatic effects on gene expression, we estimated the power through simulations. We assumed that there were *k*_*1*_ SNPs in the first gene and *k*_*2*_ SNPs in the second gene. Thus, there were totally *k*_*1*_ *k*_*2*_ SNP pairs between these two genomic regions. For the *h* ^*th*^ pair of SNPs, let ${Q}_{h_1}$ and ${q}_{h_1}$ be two alleles at the SNP in the first gene, ${Q}_{h_2}$ and ${q}_{h_2}$ be two alleles at the SNP in the second gene. Let *u*_*ijkl*_^*h*^ denote her/his genotypes of the *h* ^*th*^ pair of SNPs, where $i j\in {Q}_{h_1}{Q}_{h_1},{Q}_{h_1}{q}_{h_1},{q}_{h_1}{q}_{h_1}$ and $kl\in {Q}_{h_2}{Q}_{h_2},{Q}_{h_2}{q}_{h_2},{q}_{h_2}{q}_{h_2}$. Let ${g}_{u_{ijkl}}^h\left(\tau \right)$ denote her/his genotypic value in the *h* ^*th*^ pair of SNPs at genomic position *τ* influencing gene expressions. Then we can use the following multiple regression model to generate the function-valued trait (RNA-seq) of the *u* ^*th*^ individual of the *h* ^*th*^ pair of SNPs at the genomic position *τ*.

18 $$ {y}_u\left(\tau \right)={\displaystyle {\sum}_{h=1}^{k_1{k}_2}{g}_{u_{ijkl}}^h}\left(\tau \right)+{\varepsilon}_u\left(\tau \right), u=1,\dots, n, $$

${g}_{u_{ijkl}}^h\left(\tau \right)={\lambda}_{u_{ijkl}}^h g\left(\tau \right)$, ${\lambda}_{u_{ijkl}}^h$ is a risk parameter which is determined by the gene interaction model (Additional file 5: Table S2), the risk parameter *r* varies from 0 to 1, and *g*(*τ*) is a common genotype coefficient function fitted by the real RNA-seq data and *ε*_*u*_(*τ*) is the error stochastic process and estimated from the null model as that in null distribution in the test statistics section.

## Additional files

Additional file 1: Table S1. Summary statistics of genetic variants in the five genes. (XLS 25 kb)

Additional file 2: Table S3. A list of significant cis-trans interactions in the MAPK pathway. (XLS 114 kb)

Additional file 3: Table S4. Ten communities and their significantly enrished pathways. (XLSX 12 kb)

Additional file 4: Figure S1. A. The real RNA-seq curve of the gene *LMNB2* and the data simulated by the negative distribution of the gene *LMNB2.* B. The real RNA-seq curve of the gene *LMNB2* and the curve estimated by the FPCA of the RNA-seq data. (ZIP 120 kb)

Additional file 5: Table S2. The value of risk parameter in different interaction models. (XLS 30 kb)

## Abbreviations

BFGM: FRGM with Functional Response and Functional Predictors
FRGM: Functional regression model
NGS: Next generation sequencing
PCA: Principal component analysis
SFGM: FRGM with scalar response and functional predictors
WGS: Whole genome sequencing
