# Supplementary figures and images for: Functional regression method for whole genome eQTL epistasis analysis with sequencing data
Source: BMC Genomics. 2017 May 18;18:385. doi: 10.1186/s12864-017-3777-4 (PMC5436462; doi:10.1186/s12864-017-3777-4)

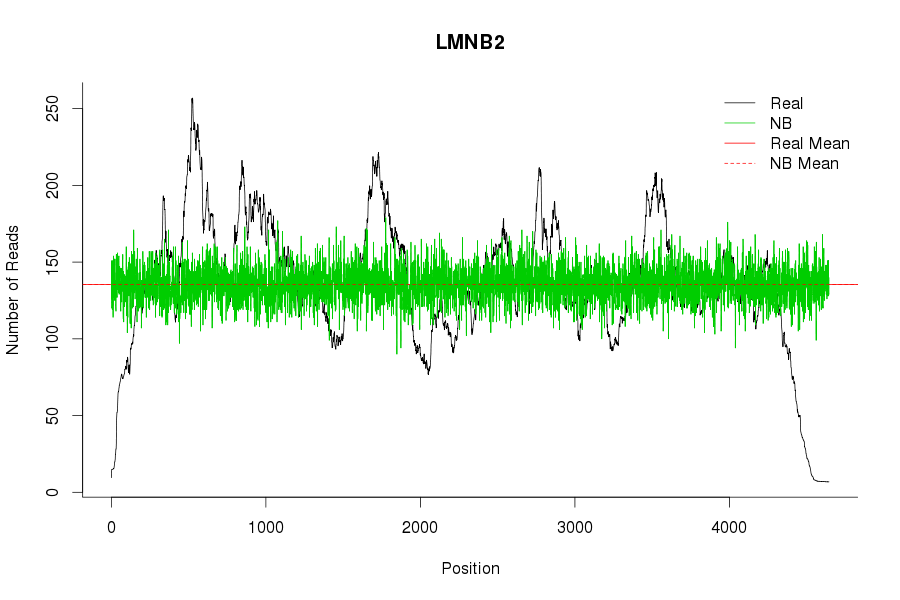

Supplement: Supplementary file 4 — A. The real RNA-seq curve of the gene LMNB2 and the data simulated by the negative distribution of the gene LMNB2. B. The real RNA-seq curve of the gene LMNB2 and the curve estimated by the FPCA of the RNA-seq data. (ZIP 120 kb) [file 12864_2017_3777_MOESM4_ESM.zip › FigureS1A.png]

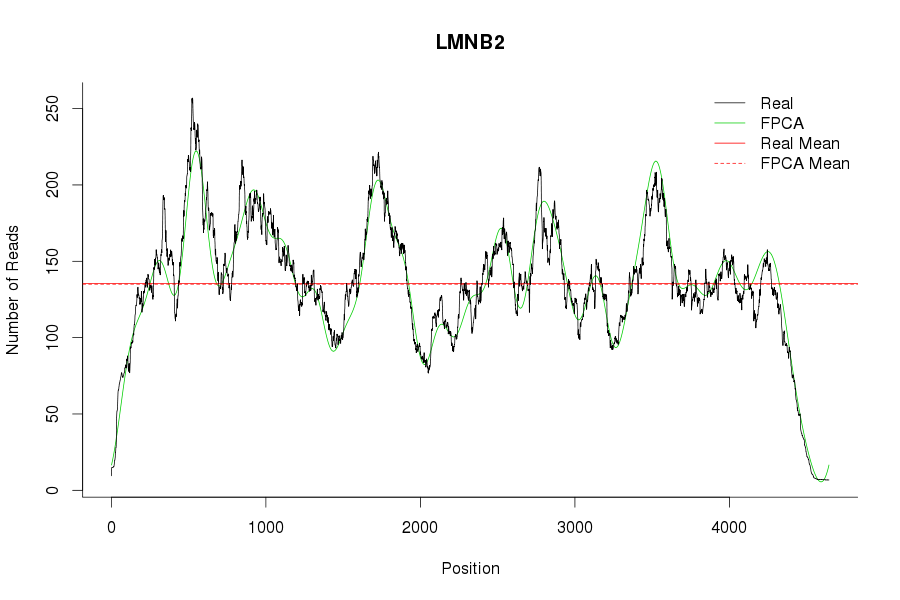

Supplement: Supplementary file 4 — A. The real RNA-seq curve of the gene LMNB2 and the data simulated by the negative distribution of the gene LMNB2. B. The real RNA-seq curve of the gene LMNB2 and the curve estimated by the FPCA of the RNA-seq data. (ZIP 120 kb) [file 12864_2017_3777_MOESM4_ESM.zip › FigureS1B (4).png]
